# Supplementary material for: Composite likelihood method for inferring local pedigrees
Source: PLoS Genet. 2017 Aug 21;13(8):e1006963. doi: 10.1371/journal.pgen.1006963 (PMC5578687; doi:10.1371/journal.pgen.1006963)
Supplement: S1 Table — (PDF) [file pgen.1006963.s001.pdf]

Types of Pairwise Relationships

This table summarizes the possible pairwise relationships we support in our method. The table below shows the "cousin" relationships, in which two individuals share one or more common ancestors. These types of relationships can be represented as the number of common ancestors the two individuals share k, the number of meiosis between the common ancestor and the first individual (m_1), and the number of meiosis between the common ancestor and the second individual m_2. Also given are the corresponding Jacquard coefficients, w_1 and w_2, and the total number of meioses between the two individuals, α.

k	m_1	m_2	α	w_1	w_2
1	1	1	2	1/2	0
1	2	1	3	1/4	0
1	2	2	4	1/8	0
1	3	1	4	1/8	0
1	3	2	5	1/16	0
1	3	3	6	1/32	0
1	4	1	5	1/16	0
1	4	2	6	1/32	0
1	4	3	7	1/64	0
1	4	4	8	1/128	0
2	1	1	4	1/2	1/4
2	2	1	5	1/2	0
2	2	2	6	1/4	0
2	3	1	6	1/4	0
2	3	2	7	1/8	0
2	3	3	8	1/16	0
2	4	1	7	1/8	0
2	4	2	8	1/16	0
2	4	3	9	1/32	0
2	4	4	10	1/64	0

Table 1: Cousin relationships

Another type of relationships is the direct ancestor-descendant relationships, such as parent-offspring. Table 2 shows such relationships, represented by the number of meioses separating the two individuals, α, and their corresponding Jacquard coefficients, w_1 and w_2.

α	w_1	w_2
1	1	0
2	1/2	0
3	1/4	0
4	1/8	0

Table 2: Ancestor-descendant relationships

Finally, we have the unrelated relationship: $w_1 = 0$ and $w_2 = 0$.
