# Supplementary figures and images for: Composite likelihood method for inferring local pedigrees
Source: PLoS Genet. 2017 Aug 21;13(8):e1006963. doi: 10.1371/journal.pgen.1006963 (PMC5578687; doi:10.1371/journal.pgen.1006963)

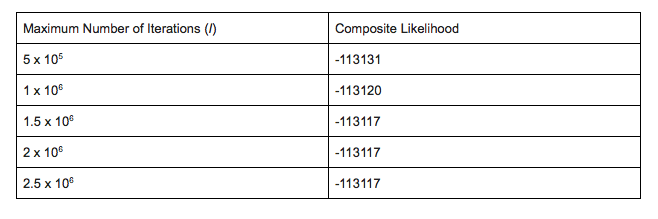

Supplement: S2 Table — Composite likelihood score at various stopping times given by I for a particular instance of simulation B. (TIFF) [file pgen.1006963.s002.tiff]

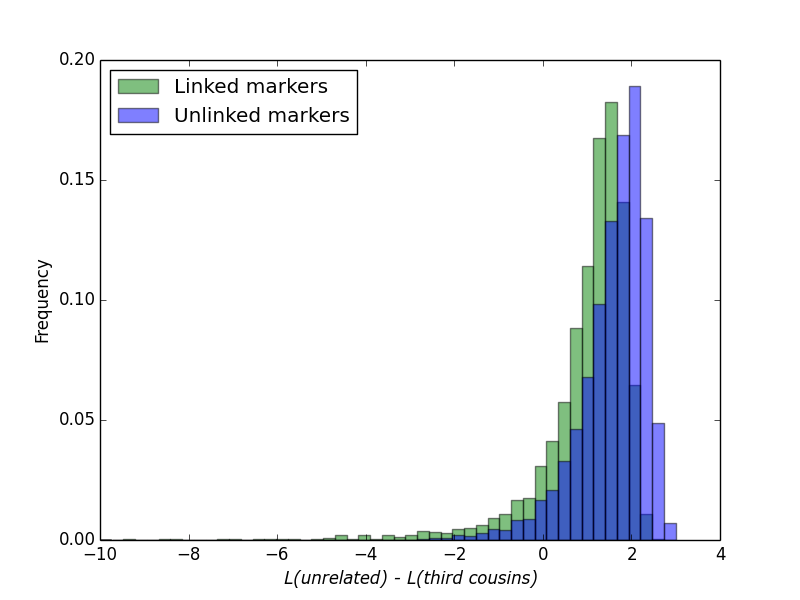

Supplement: S1 Fig — The figure shows the histogram of the log likelihood difference, L(unrelated) − L(third cousins), when the true relationship is unrelated. Unrelated pairs often have higher likelihoods for being third cousins when LD is present in the data, as shown by the histogram corresponding to linked markers. The data were simulated with msprime and the likelihoods were computed using RELATE. (TIF) [file pgen.1006963.s006.tif]

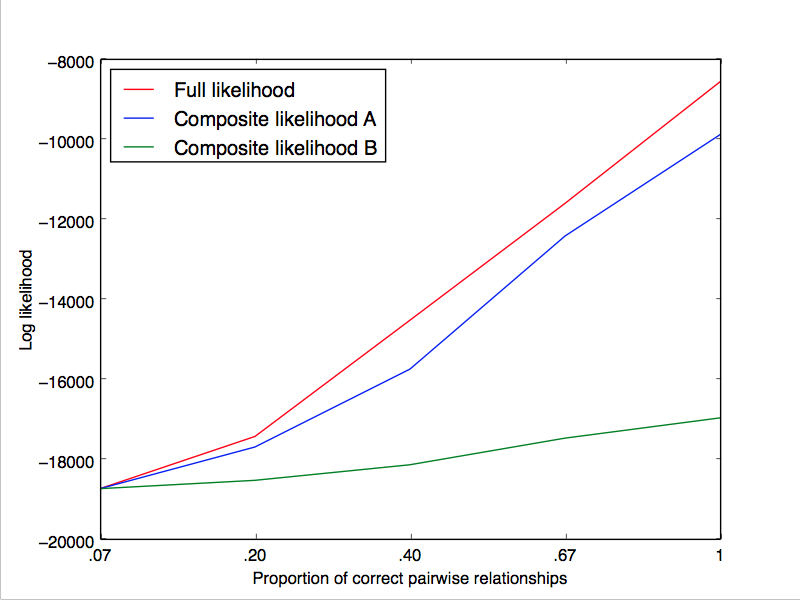

Supplement: S2 Fig — The x-axis measures how close the test pedigree is to the true pedigree; the test pedigree becomes closer to the truth from left to right. In this simulation, the composite likelihood given by Eq (1) approximates the full likelihood more closely than Eq (2). (TIFF) [file pgen.1006963.s007.tiff]

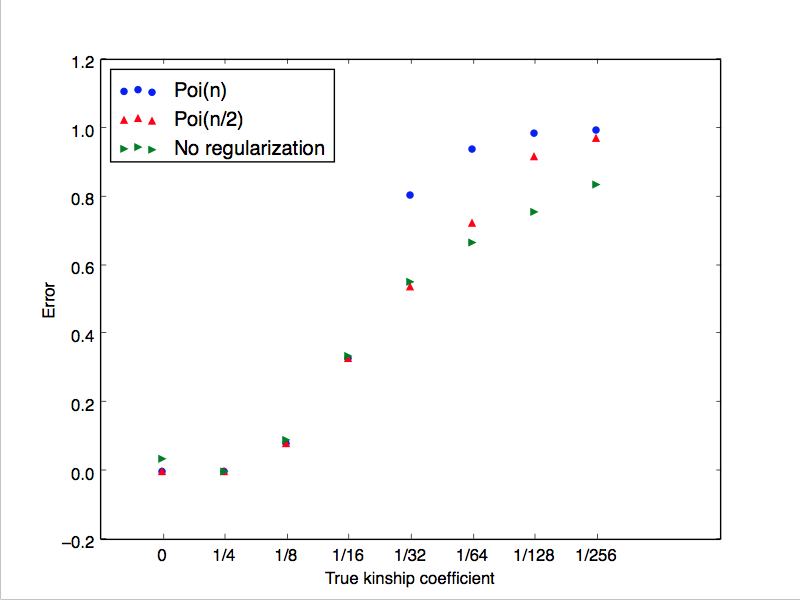

Supplement: S3 Fig — Accuracy of simulated annealing method on simulated data at 2000 markers under different levels of regularization. (TIFF) [file pgen.1006963.s008.tiff]

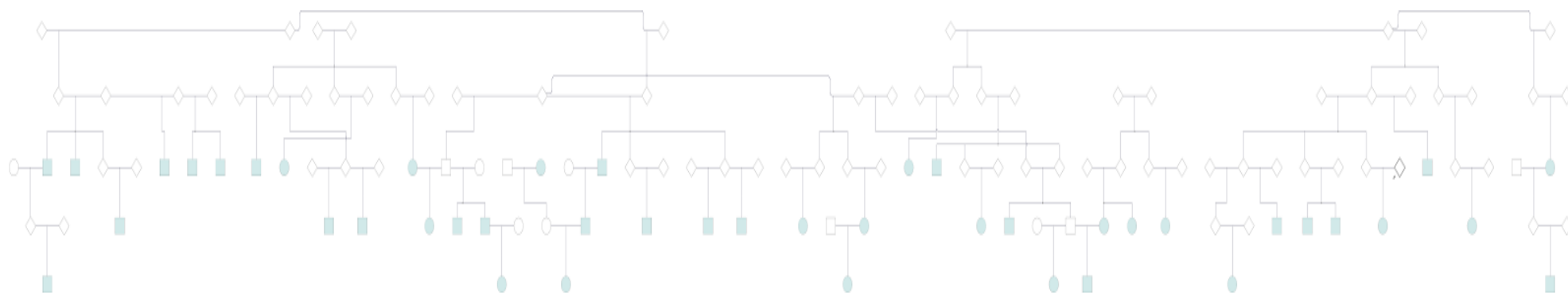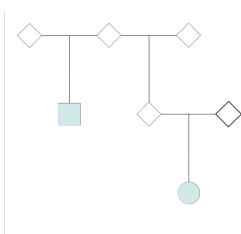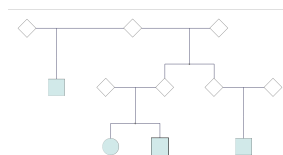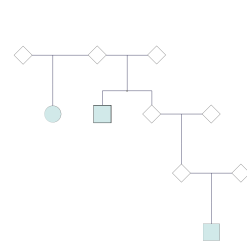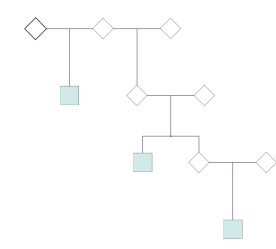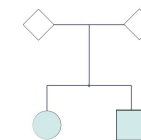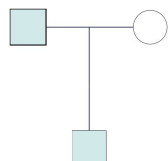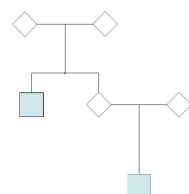

Supplement: S4 Fig — Shaded nodes indicate sampled individuals; unshaded for unsampled; squares for male; circles female; diamonds for unknown sex. (PDF) [file pgen.1006963.s009.pdf]

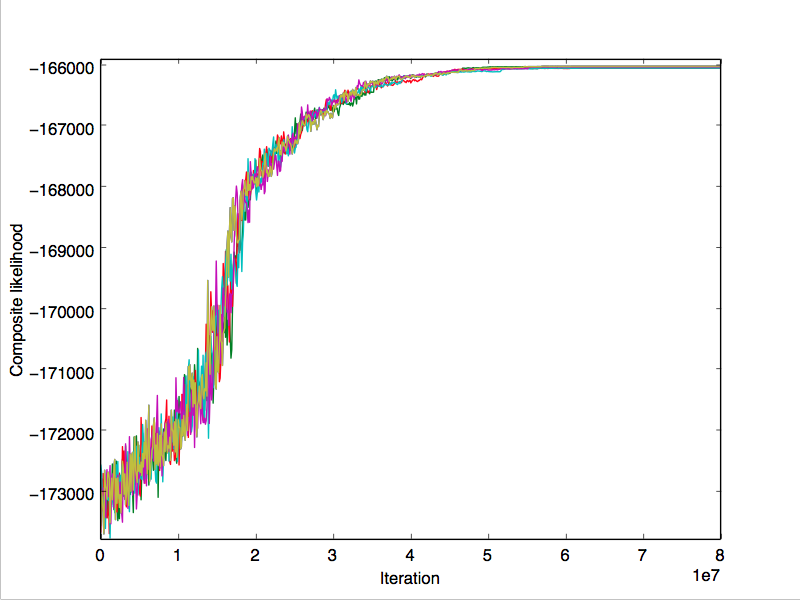

Supplement: S5 Fig — (TIFF) [file pgen.1006963.s010.tiff]
