# Supplementary material for: Composite likelihood method for inferring local pedigrees
Source: PLoS Genet. 2017 Aug 21;13(8):e1006963. doi: 10.1371/journal.pgen.1006963 (PMC5578687; doi:10.1371/journal.pgen.1006963)
Supplement: S1 Text — (PDF) [file pgen.1006963.s003.pdf]

S1 Transitions Between Pedigree Graphs

Pedigree representation

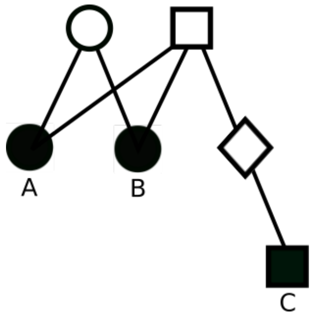

Figure 1: Pedigree graph. The shapes indicate the sex of the node (circle=female, square=male, diamond=unknown) and the color indicates whether the individual was sampled (shaded=sampled, unshaded=unsampled).

We explore the pedigree space by jumping from one pedigree graph to another. In a pedigree graph, a node represents an individual and an edge represents a parent-offspring relationship. Each node has a set of features: sex (male, female, or unknown), sample status, and depth. As shown in diagram above, the unshaded nodes represent ghost individuals for whom we do not have genotype data (unsampled); the shaded nodes represent individuals for whom we have genotype data (sampled). To represent the pedigree compactly, unsampled individuals are represented only if it connects at least two sampled individuals. For example, node C has one ghost parent that connects C to A and B. But the other parent of C is not represented since it would not be connecting two or more sampled individuals. In addition, each node belongs in a particular depth. For example, individuals A and B belong in depth 1, whereas individual C belongs in depth 0.

When we propose a new pedigree configuration, we must check whether the new configuration is a valid pedigree graph. For our study, we restrict ourselves to outbred, noncyclic pedigrees (except cycles formed by full siblings). We say that a pedigree is valid if all of the following conditions are satisfied:

1. Each node has 0, 1, or 2 parent nodes. If the node has two parents, the two parents have opposite sexes.
2. Parents of a node belong in the same generation.
3. The pedigree does not create loops except in the case of full siblings. For example, double first cousins are not allowed. Inbreeding is also not allowed.
4. The pedigree does not span more than a maximum number of generations. For this report, the maximum number of generations is 5.
5. An ancestor node is older than its descendants, if age information is available.

Transitions

This section describes the possible transitions between pedigree graphs. For each of the moves described below, we reject the move if the proposed pedigree is not a valid pedigree (See Pedigree representation). We denote nodes that represent unsampled

individuals as ”ghost nodes” and nodes that represent sampled individuals as ”sampled nodes”.

1. Link: join two pedigrees
   (a) Choose a random pair of nodes i and j, where each pair has an equal probability of being chosen.
   (b) Choose target depth d, where d is drawn from the geometric distribution. The target depth is the depth at which i and j will share a common ancestor. If the target depth exceeds the maximum depth, reject.
   (c) Choose the target sex for the would-be common ancestor, male or female, with equal probability. Follow a random path from i to reach an ancestor of the target sex at depth d. That is, starting from i, choose a male or female parent with equal probability, and move up through the pedigree until depth $d-1$ is reached; at depth d, choose the ancestor with the target sex. Repeat the process for j.
   (d) Merge the two ancestors of i and j. If the merging creates an invalid pedigree, reject.
2. Cut: detach a subpedigree.
   (a) Choose a random node i. With equal probability, choose the target sex of the parent from which i will be cut. If the parent of the chosen sex is not present in the pedigree, reject.
   (b) Remove the edge between i and the parent of the target sex.
3. Split: remove a subset of children from its parent pedigree.
   (a) Choose a random node i. Choose a random subset of i's children, where each subset has an equal probability of being chosen.
   (b) Remove the edges between the selected children and i. Make a new ghost parent for the selected children.
4. CutLink: combine Cut and Link in a single move.
5. SplitLink: combine Split and Link in a single move.
6. Contract: merge a child node with its parent node.
   (a) Choose a random node i. If i does not have exactly one ghost parent of the same sex, reject.
   (b) Remove an edge between i and its ghost parent, merging the two nodes. That is, delete the ghost parent; with equal probability, either shift the subpedigree containing i up one depth or shift the parent subpedigree down one depth; make the grandparents of i its new parents. If the merging creates an invalid pedigree, reject.
7. Stretch: Add an edge between a child and its parent.
   (a) Choose a random node i. If i does not have any parent nodes, reject.
   (b) Disconnect i from its parents; with equal probability, either shift the resulting subpedigree containing i down one depth or shirt the subpedigree containing its parent up one depth.
   (c) Make a new ghost node that has the same sex as i and make the ghost node the new parent parent of i.

(d) Make the old parents of i new parents of the ghost node. If this results in an invalid pedigree, reject the move

8. SwapDescAnc: swap ancestor and descendent.
   (a) Choose a random sampled node i.
   (b) Choose a random sampled node j among is sampled ancestors who have the same sex as i.
   (c) Swap i and j. If an invalid pedigree is generated, reject the move.
9. ShiftClusterLevel: shift the depth of family cluster
   (a) Choose a random node i and get the nodes connected to i, including i.
   (b) Choose a new target base depth d, drawn from the geometric distribution.
   (c) Adjust the depth of each node belonging to i's cluster so that the lowest depth of the cluster is d. If the shift violates the depth constraint, reject the move.
10. SwitchSex: switch the sex of an individual(s).
    (a) Choose a random node i. If i's sex is fixed (e.g. i is a sampled node and has a fixed sex, or has a spouse with a fixed sex), reject the move.
    (b) Switch i's sex.
    (c) Switch the sex of all nodes wth conflicting sexes caused by step (b). For example, if i has a spouse, the spouse must now also switch his/her sex.
11. FullSiblingsToParentOffspring: change full sibling relationship to parent-offspring relationship.
    (a) Choose a random node i. If i does not have any full siblings, reject.
    (b) Choose a random node j from the set of full siblings of i.
    (c) Disconnect i from its parents. With equal probability, either shift i's cluster down one depth or shift j's cluster up one depth.
    (d) Make j parent of i. If this results in an invalid pedigree, reject the move.
12. ParentOffspringToFullSiblings: change parent-offspring relationship to full sibling relationship.
    (a) Choose a random node i. If i does not have exactly one parent, reject the move. Call this parent p.
    (b) Detach i from p. With equal probability, shift either i's cluster up one depth or shift p's cluster down one depth.
    (c) Form a full sibling relationship between i and p. If this results in an invalid pedigree, reject.
13. FullSiblingsToSelf: merge two full sibling nodes into one node.
    (a) Choose a random node i. If i does not have any full siblings with the same sex as i, reject.
    (b) Choose a random node j among the nodes who are full siblings with i and who also have the same sex as i.
    (c) Merge i and j. If this results in an invalid pedigree, reject the move.

14. SelfToFullSiblings: split a single node into two nodes that form a full sibling relationship.

    (a) Choose a random node i.
    (b) Make a ghost node j that has the same sex as i. Set parents of j to parents of i.
    (c) Assign a random set of i's children to j. If this results in an invalid pedigree, reject.

15. HalfSiblingsToParentOffspring: change half sibling relationship to parent-offspring relationship.

    (a) Choose a random node i. If i does not have any half siblings, reject.
    (b) Choose a random node j from the set of half siblings of i.
    (c) Detach i from the parent whose sex is the same as j.
    (d) With equal probability, either shift i's cluster down one depth or shift j's cluster up one depth.
    (e) Make j parent of i. If this results in an invalid pedigree, reject.

16. ParentOffspringToHalfSiblings: change parent-offspring relationship to half sibling relationship.

    (a) Choose a random node i. If i does not have any parents, reject the move.
    (b) Choose one of i's parent at random. Call this node j.
    (c) Detach i from j. With equal probability, shift either i's cluster up a depth or shift j's cluster down a depth.
    (d) Form a half sibling relationship between i and j. If this results in an invalid pedigree, reject.

17. ParentOffspringToOffspringParent: change parent-offspring relationship to offspring-parent.

    (a) Choose a random sampled node i.
    (b) Choose a random node j among i's children.
    (c) Disconnect i from its children and shift i down two depths.
    (d) Make j new parent of i. If this results in an invalid pedigree, reject.

18. OffspringParentToParentOffspring change offspring-parent relationship to parent-offspring .

    (a) Choose a random sampled node i.
    (b) Choose a random parent of i. Call the chosen j.
    (c) Disconnect i from its parents and shift it two depths up.
    (d) Make i new parent of j. If this results in an invalid pedigree, reject.

19. UncleToNephew: change uncle-nephew relationship to nephew-uncle relationship.

    (a) Choose a random node i.
    (b) Choose a random node j among the nephews/nieces of i.
    (c) Disconnect i from its parents. With equal probability, either shift i's cluster down two depths, shift j's cluster up two depths, or shift i's cluster down one depth and j's cluster up one depth.

(d) Make a new parent (ghost node) for i. Make this ghost parent and j full siblings. If this results in an invalid pedigree, reject.

20. NephewToUncle: change nephew-uncle relationship to uncle-nephew relationship.

    (a) Choose a random node i.
    (b) Choose a random node j among the uncles/aunts of i.
    (c) Disconnect i from its parents. With equal probability, either shift i's cluster up two depths, shift j's cluster down two depths, or shift i's cluster up one depth and j's cluster down one depth.
    (d) Choose a random parent of j. Make this parent and j full siblings. If this results in an invalid pedigree, reject.

21. HalfSiblingsToGP: change half sibling relationship to grandparent-grandchild relationship.

    (a) Choose a random node i.
    (b) Choose a random node j among the half siblings of i.
    (c) Disconnect i from j. With equal probability, either shift i's cluster down two depths, shift j's cluster up two depths, or shift i's cluster down one depth and j's cluster up one depth.
    (d) Make a new parent for j (sex of parent is randomly chosen). Call this new parent p. Make j parent of p. If this results in an invalid pedigree, reject.

22. GPtoHalfSiblings: change grandparent-grandchild relationship to half sibling relationship.

    (a) Choose a random node i.
    (b) Choose at random parent node j from which i will be cut.
    (c) Choose a random node k among the parents of j.
    (d) Disconnect i from j. With equal probability, either shift i's cluster up two depths, shift j's cluster down two depths, or shift i's cluster up one depth and j's cluster down one depth.
    (e) Make i and k half siblings via a common parent whose sex is randomly chosen. If this results in an invalid pedigree, reject.
