# Supplementary material for: Composite likelihood method for inferring local pedigrees
Source: PLoS Genet. 2017 Aug 21;13(8):e1006963. doi: 10.1371/journal.pgen.1006963 (PMC5578687; doi:10.1371/journal.pgen.1006963)
Supplement: S2 Text — (PDF) [file pgen.1006963.s004.pdf]

S2 Command Lines for Running External Softwares

1. To run PLINK to estimate population allele frequencies:
   plink –tfile 100founders –freq –out 100founders,
   where100founders is the name of tped and tfam files containing 100 unrelated diploid individuals. This generates 100founders.frq that contains allele frequency estimates.

2. To run PLINK to estimate IBD proportions:
   plink –tfile testFile –genome –read-freq 100founders.frq –out testFile,
   where testFile is the name of tped and tfam files containing test samples and 100founders.frq are allele frequency estimates from above. This generates testFile.genome containing IBD proportion estimates.

3. To run RELATE to estimate IBD proportions and IBD segments:
   relateHMM -g geno -p pos -c chr -o options -d indiv -post postout -k kout,
   where geno is the genotype file containing test individuals and unrelated founder individuals. The unrelated founders are only used to estimate the allele frequencies and haplotype frequencies. Details for the remaining files can be found in the RELATE manual (see main text for citation). The parameters in the option file are shown below:

   1 #1=allpairs 0=normal run
   0 #pair[0]
   1 #pair[1]
   0 #double recombination 0 #LD=0=rsq2 LD=1=D
   0.001 # alim[0]
   5 # alim[1]
   0 # doParameter calculation (pars)
   0 # par[0] = a this is only used if doParameter is set to 1
   0 # par[1] = k2 this is only used if doParameter is set to 1
   0 # par[2] = k1 this is only used if doParameter is set to 1
   1 # ld_adj
   0.01 # epsilon
   1 # back
   0 # doPrune
   0 # prune_value
   0 # fixA
   0.0 # fixA_value
   0 # fixK2
   0.0 # fixk2_value
   0 # calculateA
   0.013 # phi_value
   0.1 # convergence_tolerance
   5 # times_to_converge
   10 # times_to_run
   2 # back2

4. To run PRIMUS:
   run_PRIMUS.pl -p IBDfile –sex_file sexFile –plink_ex myPlinkPath –no_PCA_plot –no_IMUS -o myOutPath –max_gens 5,
   where IBDfile contains IBD proportion estimates from either PLINK or RELATE; sexFile contains sex information.

5. To run ERSA:

ersa –control_files=controlFile –segment_files=segmentFile –model_output_file=ersaModelFile –output_file=ersaResultFile–confidence_level .95 –rec_per_meiosis 39,
where controlFile contains IBD segment estimates by RELATE for 100 founder individuals; segmentFile contains IBD segment estimates for the test samples.

6. To run PADRE:
run_PADRE.pl –ersa_model_output ersaModelFile –ersa_results ersaResultFile –project_summary summaryFile –degree_rel_cutoff 3 –output_dir,
where the file paths point to appropriate output directories for ERSA and PRIMUS.
