# Supplementary material for: Composite likelihood method for inferring local pedigrees
Source: PLoS Genet. 2017 Aug 21;13(8):e1006963. doi: 10.1371/journal.pgen.1006963 (PMC5578687; doi:10.1371/journal.pgen.1006963)
Supplement: S3 Text — Summarizes a simulation study that shows how CLAPPER performs on inbred pedigrees. (PDF) [file pgen.1006963.s005.pdf]

# S3 Effects of Inbreeding

The current version of CLAPPER does not support inbred pedigrees and rejects any such pedigrees during its search algorithm. To test how CLAPPER performs on inbred pedigrees, we simulated two inbred individuals who form a first cousin relationship. In the first scenario, the two individuals are inbred via their grandparents who form a first cousin relationship (Fig 1A). In the second scenario, the grandparents form a full sibling relationship (Fig 1B).

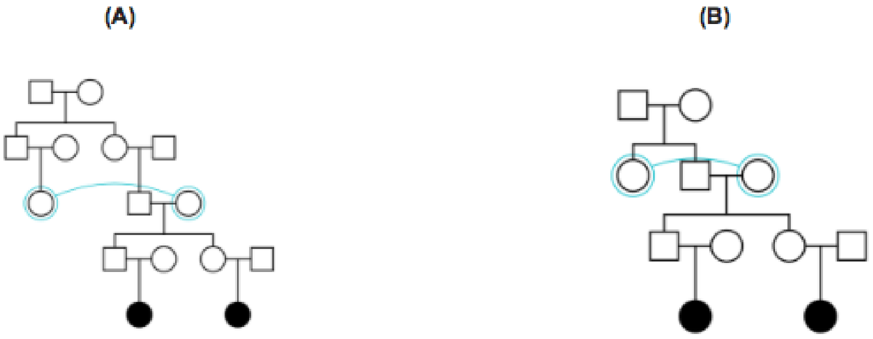


Figure 1: Simulated inbred pedigrees. The shaded nodes indicate sampled individuals and unshaded nodes indicate unsampled individuals. The nodes connected by a blue line indicate that the two nodes are the same. (A) Two sampled first cousins whose grandparents are first cousins. (B) Two sampled first cousins whose grandparents are full siblings.

For each scenario, we ran our method on 100 replicate simulations. We allowed the two sampled nodes to be in any generation. Fig 2 shows four different types of pedigrees inferred by our method for the first scenario. In all of these pedigrees, the two sampled individuals have the kinship coefficient of 1/16, which is equal to the kinship coefficient of first cousins.

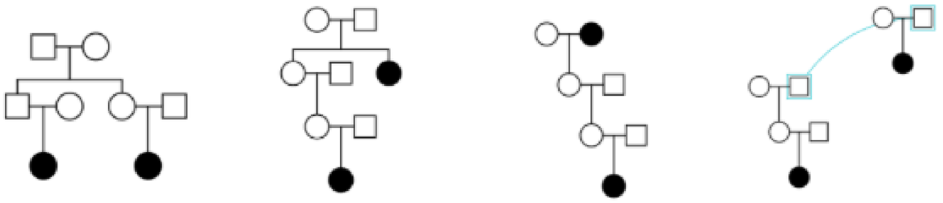

Figure 2: Different types of pedigrees inferred by CLAPPER for the pedigree shown by Fig 1A.

Fig 3 shows the inferred pedigree for the second scenario. Due to inbreeding, our method inferred that the two individuals had a uncle-nephew relationship, which is one degree of relationship closer than the truth.

These simulations show that although CLAPPER does not consider inbred pedigrees, it estimates relationships close to the truth. When the level of inbreeding is

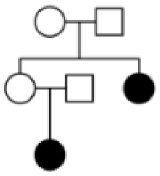

Figure 3: Different types of pedigrees inferred by CLAPPER for the pedigree shown by Fig 1B.

relatively low such as that shown by Fig 1A, the degree of relationship is correctly inferred by CLAPPER. However, when the level of inbreeding is very high such as that shown by Fig 1B, CLAPPER estimates that the relationship is closer than the true relationship.
